# Association between latent profile of dietary intake and cardiovascular diseases (CVDs): Results from Fasa Adults Cohort Study (FACS)

**DOI:** 10.1038/s41598-023-44766-4

**Published:** 2023-10-18

**Authors:** Mohammad Ariya, Mehdi Sharafi, Sima Afrashteh

**Affiliations:** 1 Noncommunicable Diseases Research Center, Fasa University of Medical Sciences, Fasa, Iran; 2 Department of Nutrition, Fasa University of Medical Sciences, Fasa, Iran; 3 Social Determinants in Health Promotion Research Center, Hormozgan Health Institute, Hormozgan University of Medical Sciences, Bandar Abbas, Iran; 4 Department of Biostatistics and Epidemiology, Faculty of Health and Nutrition, Bushehr University of Medical Sciences, Bushehr, Iran

**Keywords:** Diseases, Health care, Medical research

## Abstract

Cardiovascular diseases (CVDs) have been among the most significant non-communicable diseases. Dietary risks account for the most cause of CVDs mortalities. Evaluating overall dietary patterns (through the Latent profile of dietary intake) can provide a more accurate prediction regarding the prevalence of CVDs. The present cross-sectional study aimed to investigate the relationship between the latent profile of dietary intake and CVDs prevalence. The population of the Fasa Adults Cohort Study was employed to gather the data (n = 8319). A modified FFQ was employed to assess eating behaviors. Minerals, as well as the energy intake and total fiber, were measured using Nutritionist IV software (version 7.0). To estimate the prevalence of CVDs, accurate records of patients' histories were made. Individuals were clustered according to their dietary intake using latent profile analysis. The mean age was 48.75 ± 9.59 years, and 53.28% (4430) were women. 63.9% of participants with low Socioeconomic Status (SES) were in the low-intake profile (*P* < 0.001), and high SES increases the odds of being in the high-intake profile (OR_high/low_ = 2.87, 95% CI 2.55–3.24). The low-intake group had the lowest amount of physical activity (Met) (*P* < 0.001). The result of multivariate logistic regression revealed that categorized in the low-intake group significantly increased the development of CVDs (OR = 1.32, 95% CI 1.07–1.63, *P* = 0.010). The mean micronutrients and total fiber, in individuals with a low intake profile, were significantly lower than other groups (*P* < 0.001). Overall, we estimated that a low intake of all food groups increases the odds of developing CVDs significantly.

## Introduction

Cardiovascular diseases (CVDs) have been among the most significant non-communicable diseases (NCDs) from 1980 to the present and have been linked to numerous mortalities, according to the Global Burden of Disease (GBD) research^1^. Investigations have identified a number of reasons for the development of CVDs, and GBD estimated in 2017 that dietary risks account for 55% of mortalities and 60% of disability-adjusted life years in low-and middle-income countries^2^. According to other researches, CVDs are the primary cause of NCDs deaths worldwide, making it the second leading cause of years of life lost in developing nations^3,4^.

The prevalence of non-communicable diseases is significantly influenced by diet. As a result, around 500,000 mortalities are attributable to poor diet in the US each year, with 48% of them being caused by CVDs^5^. In this regard, it is important to mention that low-income communities are primarily affected by the detrimental effects of inadequate diet^6^. As a result, gaining a thorough understanding of dietary patterns, nutritional deficiencies, and the latent profile of dietary consumption may help to some extent prevent the onset of non-communicable diseases like CVDs.

Although research indicates that dietary classifications like the Mediterranean Diet and the Healthy Eating Index are linked to a decrease in the prevalence of chronic diseases, including CVDs, these classifications also have restrictions, such as the fact that they offer various definitions of what constitutes a healthy diet^7^. Their explanation of heterogeneous dietary patterns is likewise described in this classification in quite different ways^7,8^. As a result, it would appear that we require a more thorough index to explain the connection between non-communicable diseases and diet.

Furthermore, despite some studies demonstrating a direct correlation between a healthy diet and reduced CVDs incidence, inconsistencies persist within the scientific literature^2,9^. Moreover, the majority of research that evaluated the relationship between diet and CVDs without considering synergistic effects on health outcomes was restricted to particular food groups or patterns^10,11^. Therefore, it appears that evaluating overall dietary patterns instead of focusing on specific diets can provide a more accurate prediction regarding dietary patterns and the prevalence of diseases^12^. To better define the incidence of multi-component constructs like CVDs and approximate its nutritional risk factors in the target population, the estimation of the Latent profile of dietary intake was developed^13^.

The Latent profile of dietary intake can be a useful tool to identify the dietary pattern and offer a more precise estimate of the prevalence of non-communicable diseases in the target population, even though there are limited investigations in this area^7^. According to our investigations, this is the first study in this field to have been carried out in Iran. The results of all prior investigations, which did not examine the overall dietary pattern and only examined a specific dietary pattern in relation to the incidence of CVDs, were inconclusive^14–18^. Consequently, the current study endeavors to unravel the latent profile of dietary intake and its intricate association with CVDs prevalence in the cohort population of Fasa University of Medical Sciences because of the limited number of articles published worldwide in this field. Indeed, it aims to contribute valuable insights into the complex relationship between diet and non-communicable diseases.

## Result

The baseline characteristics of the study population were analyzed according to gender (Supplementary Table No. 1). The mean age of the study population was 48.75 ± 9.59 years (*n* = 8319), and most of them were female (4430; 53.28%). Moreover, 691 of participants (8.30%) were smoking, and 987 of them had CVDs (11.86%). Furthermore, based on the characteristics of the study population, we observed that there were significant differences between Socioeconomic Status (SES), smoking and drug use, having CVDs, energy intake, physical activity (MET), and body mass index (BMI) among males and females (*P* < 0.001) (Supplementary Table No. 1).

In the current study, latent profile analysis (LPA) was implemented for 2 to 5-class models. Class 2 had the highest AIC, BIC, and Consistent Akaike information criterion (CAIC) values, suggesting that these models were less suitable for data than models 3, 4, and 5. Based on the findings, although model 3 had more AIC and BIC than classes 4 and 5, the entropy of this model was higher. Therefore, we chose model 3 because it was concise and had better interpretability than the other two classes. (Table 1).

**Table 1:** Model fit statistics for latent profile analysis by number of estimated profiles.

Class	LL	AIC	CAIC	BIC	SABIC	Entropy	Prob. Min.	Prob. Max.
2	− 292,840	585,874	586,653	586,556	5.86e5	0.87	0.92	0.98
3	− 284,680	569,621	570,664	570,534	5.70e5	0.94	0.93	0.99.9
4	− 282,091	564,507	565,816	565,653	5.65e5	0.89	0.91	0.99.9
5	− 280,565	561,523	563,096	562,900	5.62e5	0.91	0.90	0.99

*LL* Log-likelihood, *AIC* Akaike information criterion, *BIC* Bayesian information criterion, *CAIC* Consistent Akaike information criterion, *SABIC* Sample size-adjusted Bayesian information criterion, *Entropy* A measure of classification uncertainty, *Prob. Min.* Minimum of the diagonal of the average latent class probabilities for most likely class membership, by assigned class, *Prob. Max.* Maximum of the diagonal of the average latent class probabilities for most likely class membership, by assigned class. The maximum should also be as high as possible, reflecting greater classification certainty.

As shown in Table 2, the first profile consisted of 8.9% (741 persons) and was characterized by "Low Intake" values. In this class, individuals reported a very low intake of whole grains, leafy vegetables, cruciferous vegetables, potatoes, and, other foods. The second class includes 44.6% (3718 persons) of the sample. Overall, members of this profile had the highest mean for intake of whole grains, red meat and processed foods, oil and fat groups, ungrouped foods, vegetables, fruits, and legumes. We called this profile "High Intake". The third profile was the biggest, corresponding to 46.4% (3860 persons) of the sample, and included a moderate intake of whole grains, cruciferous vegetables, tomatoes, legumes, leafy vegetables, and other foods. We called this profile "Moderate intake". ANOVA test indicated that the mean intake of these foods was significantly different between profiles except for dark yellow vegetables (*P* < 0.001). It is noteworthy to mention that the classification of different food groups according to FFQ has been shown in Supplementary Table No. 2.

**Table 2:** Average servings per day of food items by latent dietary profile in 35–70 years old population of Fasa Persian Cohort Study (n = 8319).

Servings per day of food items (g/day)	Class 1 (low Intake) 741 (8.91%)	Class 2 (high Intake) 3718 (44.69%)	Class 3 (moderate intake) 3860 (46.40%)	F	*P* value*
	Mean ± SD	Mean ± SD	Mean ± SD		
Whole grains versus Refined grain					
Whole grains	0.77^a^ ± 1.27	1.36^c^ ± 1.68	0.97^b^ ± 1.39	160.39	< 0.001
Refined grains	2.66^**a**^ ± 0.22	2.80^**c**^ ± 0.19	2.74^**b**^ ± 0.21	171.55	< 0.001
Vegetables, fruits and legumes					
legumes	1.24^a^ ± 0.48	1.68^c^ ± 0.29	1.49^b^ ± 0.30	744.11	< 0.001
Leafy vegetables	0.51^a^ ± 0.95	1.38^c^ ± 0.40	1.06^b^ ± 0.49	294.12	< 0.001
Crucifeerois vegetables	0.34^a^ ± 1.60	1.76^c^ ± 0.86	0.83^b^ ± 1.31	58.60	< 0.001
Tomatoes	1.97^a^ ± 0.52	2.34^c^ ± 0.29	2.20^b^ ± 0.32	431.71	< 0.001
Other vegetables	1.88^a^ ± 0.49	2.36^c^ ± 0.24	2.15^b^ ± 0.27	592.74	< 0.001
Dark yellow vegetables	1.69^a^ ± 0.46	1.69^a^ ± 0.47	1.69^a^ ± 0.43	0.05	0.95
Potatoes	0.55^a^ ± 1.56	1.41^c^ ± 0.59	1.22^b^ ± 0.79	350.95	< 0.001
Summer spring fruits	1.24^a^ ± 0.71	2.17^c^ ± 0.36	1.83^b^ ± 0.43	567.55	< 0.001
Winter autumn fruits	1.13^a^ ± 0.82	1.99^c^ ± 0.39	1.68^b^ ± 0.43	425.78	< 0.001
All season fruits	1.27^a^ ± 0.81	2.13^c^ ± 0.35	1.83^b^ ± 0.42	560.90	< 0.001
Red meat and processed foods					
Red meat	0.15^a^ ± 1.34	1.02^c^ ± 0.65	0.65^b^ ± 0.83	687.23	< 0.001
Processed meats	0.28^a^ ± 0.73	0.85^c^ ± 1.10	0.55^b^ ± 0.91	150.31	< 0.001
Pizza	0.06 ± ^a^0.74	0.16^c^ ± 1.61	0.07^b^ ± 0.97	115.45	< 0.001
French fried	0.06^a^ ± 0.92	0.31^c^ ± 1.70	0.1^b^ ± 1.33	224.61	< 0.001
Snacks	0.08^a^ ± 1.12	0.38^c^ ± 1.60	0.15^b^ ± 1.47	182.22	< 0.001
Sweets	0.72^a^ ± 1.39	3.03^c^ ± 0.52	1.85^b^ ± 0.75	447.50	< 0.001
Mayonnaise and creamy dressing	0.10^a^ ± 1.16	0.72^c^ ± 1.12	0.29^b^ ± 1.36	253.43	< 0.001
Oil and fat groups					
Butter/margarine/oil	1.09^a^ ± 0.54	1.30^c^ ± 0.31	1.22^b^ ± 0.34	130.60	< 0.001
Olive oil	0.07^a^ ± 0.95	0.28^b^ ± 1.39	0.10^a^ ± 1.19	107.54	< 0.001
Nuts	0.39^a^ ± 1.25	1.87^c^ ± 0.48	1.07^b^ ± 0.73	430.36	< 0.001
Ungrouped foods					
Poultry	0.74^a^ ± 1.09	1.43^c^ ± 0.54	1.17^b^ ± 0.66	384.87	< 0.001
Organ meal	0.31^a^ ± 0.75	0.93^c^ ± 1.06	0.637^b^ ± 0.86	275.26	< 0.001
Eggs	0.39^a^ ± 1.61	1.34^c^ ± 0.72	1.07^b^ ± 0.97	323.27	< 0.001
Fish and sea food	0.13^a^ ± 0.67	0.96^c^ ± 1.11	0.51^b^ ± 0.84	406.53	< 0.001
Low and High fat dairy	1.73^a^ ± 0.75	2.33^c^ ± 0.30	2.10^b^ ± 0.40	427.41	< 0.001
Fruit juice	0.08^a^ ± 1.48	1.30^c^ ± 0.54	0.88^b^ ± 0.81	339.06	< 0.001
Condiments	0.91^a^ ± 0.53	1.65^c^ ± 0.31	1.36^b^ ± 0.37	649.04	< 0.001
Tea	2.58^a^ ± 1.16	2.73^b^ ± 0.73	2.71^b^ ± 0.79	11.19	< 0.001
Energy drink	0.19^a^ ± 1.81	1.76^c^ ± 0.80	1.17^b^ ± 1.21	318.92	< 0.001
Coffee	0.64^a^ ± 0.84	0.23^b^ ± 1.53	0.08^a^ ± 1.03	104.58	< 0.001

*All measurements transferred to log transformation.

^a,^^b,c^Mean values in rows with non-similar superscript letters(a,b,c) were significantly different.

*Test* one-way ANOVA, *Post hoc test* Tukey’s method.

The highest mean age (53.42 ± 9.95 years) was in the low-intake profile (*P* < 0.001). 58.8% of women versus 41.1% of men were in the moderate-intake profile. Based on the results, women were significantly more likely than men to membership in low-intake profile (*P* < 0.001). Most of the individuals who used cigarettes or drugs significantly were in the high-intake profile, 32.1% and 26.3% respectively (*P* < 0.001). In addition, 63.9 percent of people with low socioeconomic status (SES) were in the low-intake profile (*P* < 0.001). The mean energy intake in the high-intake profile (2617.38 ± 694) was significantly higher than those in the low and moderate groups (*P* < 0.001). Furthermore, according to BMI and physical activity (Met), the low-intake group had the least amount of BMI as well as the lowest amount of physical activity (Met), and these differences were significant (*p *< 0.001) (Table 3).

**Table 3:** Respondent characteristics by latent dietary profile in 35–70 years old of population of Fasa Adults Cohort Study (FACS).

Categorical measures	Subgroup	Medium intake	High intake	Low intake	*p* value
		N (%) *	N (%) *	N (%) *	
Sex	Male	1589 (41.17)	2064 (55.51)	234 (31.58)	< 0.001**
	Female	2271 (58.83)	1654 (44.49)	507 (68.42)	
Socio Economic Status (SES)	Low	1508 (39.08)	791 (21.29)	474 (63.97)	< 0.001**
	Medium	1358 (35.19)	1226 (32.99)	187 (25.24)	
	High	993 (25.73)	1699 (45.72)	80 (10.80)	
Smoking	No	2903 (75.25)	2521 (67.86)	564 (76.11)	< 0.001**
	Yes	955 (24.75)	1194 (32.14)	177 (23.89)	
Use Drugs	No	3139 (81.32)	2736 (73.63)	634 (85.68)	< 0.001**
	Yes	721 (18.68)	980 (26.37)	106 (14.32)	

Continues measurement	–	Mean ± SD	Mean ± SD	Mean ± SD	
Age	–	49.84 ± 9.55	46.69 $\pm$ 9.04	53.42 ± 9.95	< 0.001***
Energy intake	–	2505.81 ± 79.86	2617.38 ± 694.79	2383.51 ± 718.54	< 0.001***
Physical activity (MET)	–	41.74 ± 11.26	41.64 ± 11.91	39.58 ± 9.73	< 0.001***
BMI	–	25.45 ± 4.85	25.95 ± 4.81	24.67 ± 5.03	< 0.001***

*Within column relative frequencies.

**p value for chi-square test.

***p value for one-way ANOVA test, Post hoc test: Tukey’s method.

*BMI* Body mass index.

A multinomial logistic regression analysis adjusted for age, gender, SES, smoking, and drug use showed older age increases the odds of being in the profile of low-intake (OR = 1.03, 95% CI 1.02–1.04) compared to the moderate group. The odds of membership in the profile of low-intake (OR _female/male_ = 1.43, 95% CI 1.15–1.78) were significantly higher in women than men compared to the moderate-intake profile. Based on the findings, high SES increases the odds of being in the profile of high-intake (OR_high/low_ SES = 2.87, 95% CI 2.55–3.24) and 69% reduces the odds of being in the profile of low SES (OR_high/low_ SES = 0.29, 95% CI 0.22–0.37) compared to the moderate group (Table 4).

**Table 4:** Summary findings of multinomial logistic regression for association between latent profile of dietary intake and some of its risk factors.

Variable	Latent profile of dietary intake in 35–70 years old in FACS		
	Medium intake	High intake	Low intake
	Odds ratio (95% CI)*		
Age	1	0.96 (0.96–0.97)	1.03 (1.02–1.04)
Sex			
Male	1	1	1
Female	1	0.66 (0.58–0.74)	1.43 (1.15–1.78)
Socioeconomic			
Low	1	1	1
Moderate	1	1.61 (1.43–1.81)	0.45 (0.38–0.55)
High	1	2.87 (2.55–3.24)	0.29 (0.22–0.37)
Smocking			
No	1	1	1
Yes	1	1.11 (0.96–1.27)	1.15 (0.90–1.45)
Use Drugs	1		
No	1	1	1
Yes	1	1.07 (0.92–1.24)	0.94 (0.70–1.25)

*p* value less than 0.05 considered significant.

*The proportional odd for ordered logistic regression was violated (*p* value for chi-square test < 0.001) so, we run the multinominal logistic regression.

The result of multivariate logistic regression revealed in Table 5. Based on this regression, categorized in low latent profile (low-intake group) significantly increased developing of CVDs (OR_low intake/moderate intake_ = 1.32, 95% CI 1.07–1.63, *P* = 0.010). This analysis also showed that people in the high intake profile have a 14% (OR_high intake/moderate intake_ = 0.86, 95% CI 0.74–1.01, *P* = 0.075) lower odds of developing CVDs compared to the moderate-intake group. Although the relationship was not significant.

**Table 5:** Multivariate logistic regression for assessment of relationships between latent profile of dietary intake and prevalence of cardiovascular disease.

Variable	Adjusted odds ratio	95% CI for odds ratio	*p* value
Medium intake	1	–	–
High intake	0.86	0.74–1.01	0.075
Low intake	1.32	1.07–1.63	0.010

Dependent variable: having cardiovascular disease.

Odds Ratio adjusted for age, sex, socio-economic status, smoking and drug use.

The ANOVA test was used to compare micronutrients and total fiber between latent profiles (Table 6). Overall, the mean of all micronutrients and total fiber studied in the high intake profile was significantly higher than the low and moderate profiles (*P* < 0.05). Moreover, the mean calcium, zinc, iron, sodium, selenium, potassium, magnesium, fibers, manganese, and copper in individuals with a low intake profile was significantly lower than in the moderate and high intake groups (*P* < 0.05).

**Table 6:** Comparison of micronutrients and fiber between latent profile of dietary intake in FACS.

Variable	Medium intake	High intake	Low intake	F	*p* value*
	Mean ± SD	Mean ± SD	Mean ± SD		
Micronutrient					
Calcium	1405.08 ± 684.50	1441.95 ± 688.22	1349.71 ± 685.64	6.59	0.001
Magnesium	389.25 ± 151.34	413.763 ± 159.12	364.18 ± 146.30	43.27	0.001
Zinc	11.35 ± 4.74	12.08 ± 4.89	10.76 ± 4.53	34.57	< 0.001
Cupper	1.88 ± 0.79	2.01 ± 0.82	1.75 ± 0.71	42.30	< 0.001
Manganese	4.73 ± 2.46	4.99 ± 2.44	4.37 ± 2.45	23.91	< 0.001
Selenium	147.22 ± 76.35	151.81 ± 75.81	143.31 ± 75.22	5.67	0.003
Sodium	4691.02 ± 1975.29	4869.23 ± 2027.21	4485.25 ± 1938.26	14.82	< 0.001
Potassium	3841.28 ± 1570.03	4134.97 ± 1706.53	3528.97 ± 1443.18	57.69	< 0.001
Iron	23.45 ± 11.03	24.23 ± 11.13	22.57 ± 10.89	9.06	0.0001
Fiber					
Total Fiber	30.56 ± 13.19	32.82 ± 14.31	28.45 ± 12.56	44.57	< 0.001

*p value for one-way ANOVA test.

## Discussion

The participants in the current study were divided into three groups using LPA based on their consumption of various food groups. The most significant aspect is that participants in the low-intake group consumed significantly fewer servings of almost all food groups than the other two groups, including healthy foods or foods recognized as supporting health and well-being, such as whole grains, legumes, fruits, and vegetables, in addition to unhealthy foods like pizza, energy drinks, and other processed foods. This was also evident for the high-intake group, which used the most of practically all food groups. The CVDs prevalence was ultimately shown to be 1.3 times higher in persons classified into the low-intake category than in other groups. In other words, we estimated that a low intake of all food groups increases the risk of developing CVDs significantly. Moreover, we observed that participants with low SES consumed fewer food items, as a result, they were categorized in the low-intake group. As already mentioned, this group was more likely to develop CVDs in the current study.

One possible explanation for the increasing chance of developing CVD in the low latent profile class is that participants in this group consumed much less of all food groups, such as vegetables, fruits, and legumes, as well as red meat, than the other two groups. As a result, it may be claimed that this population is susceptible to nutritional deficiencies and poor health^7^. On the other side, individuals with low latent profiles were simultaneously identified as having poor socioeconomic positions, which limits their access to food. All of the factors mentioned above lead to a decrease in the intake of micronutrients in this population, as indicated in Table No. 6. As a result, there is a greater probability that they may experience nutritional deficiencies, which can increase CVDs prevalence.

Concerning SES, Bishop et al.'s study reported results in line with the current investigation^7^. It was also noted in a different study that those with lower SES have a much higher likelihood of developing CVDs^19^. Participants living in socioeconomically deprived areas had higher rates of CVDs and death, according to other research that also found results consistent with the findings of the current study^20–22^. The cause of this issue may be that those with low SES are more likely to have poor diets, smoke, or engage in minimal physical activity, all of which enhance the risk of developing CVDs^22^.

Furthermore, numerous studies demonstrate that adequate physical activity can either prevent or delay the CVDs outbreak^23,24^. Participants in the low-intake category in the present investigation had lower physical activity levels and a higher risk of CVDs. Furthermore, as already indicated, our findings revealed that those in the low SES category were substantially more likely to fall into the low intake category. In this regard, a review study revealed that the importance of physical activity as a possible component for preserving health is widely disregarded in low SES populations^25^. Perhaps the origin of this problem lies in the fact that individuals with low SES do not set aside adequate time for physical activity as a result of their long workdays^26^. In fact, physical activity can significantly reduce the risk of CVDs through mechanisms like triglyceride reduction, HDL increase, and decrease in coronary artery calcium^23^.

Participants in the low-intake group in the present investigation had lower BMIs than those in the other two groups. A review study revealed that normal weight obesity referred to as a normal BMI and a high percentage of fat simultaneously, can be associated with a wide range of metabolic diseases, including cardiometabolic dysfunction, although numerous investigations indicate that an increase in BMI may be regarded as a risk factor for CVD^27^. According to this study's findings, people with coronary artery disease who also have central obesity and a normal BMI had a considerably higher mortality rate^27^. In light of the preceding, these individuals may have abdominal obesity or a higher percentage of fat, which would explain why in the current study, those who were categorized as low intake had a lower BMI and a higher risk of CVDs. It is suggested that future research examine body composition to explain this relationship.

Our research demonstrated that lower intake of all food groups (healthy and unhealthy) substantially elevated the CVDs prevalence in the population studied, despite some studies showing that a healthy diet (high consumption of fruits, vegetables, legumes, chicken meat, and soy protein along with less consumption of fast food, sweets, butter, saturated oils, ghee, eggs, whole dairy products, and red meat) substantially decreases the CVDs prevalence^13^. The results of this study suggest that malnutrition in the low-intake group may be the root of this problem. Malnutrition is defined as a situation in which there is a lack of nutrition intake or uptake, which can result in an impaired clinical outcome of the disease^28^. Additional investigations have found that malnutrition in patients with all types of CVDs is linked to longer hospitalization, greater mortality, and lower survival rate^29–31^. Furthermore, the World Health Organization (WHO) indicated low fruit and vegetable intake is strongly associated with NCDs development and mortality^32^.

Higher fiber consumption, on the other hand, has been shown in studies to help reduce cardiovascular disease^33^. Participants classified as low intake in the current study had substantially lower fiber consumption. By lowering cholesterol, blood pressure, and inflammatory, increased fiber consumption can effectively prevent CVDs^34,35^. In other words, it is possible to significantly lower the risk of developing atherosclerosis and other CVDs, as well as the mortality brought on by these conditions, by increasing the amount of whole grains, fruits, and vegetables in the diet as a source of fiber^34–36^. Higher fiber consumption is linked to a considerable reduction in C-reactive protein (CRP) levels, and lower CRP levels are linked to a decreased risk of developing different kinds of CVDs^36^.

According to the current study, individuals classified in the low-intake group consumed much less fiber and different minerals, including calcium, magnesium, zinc, copper, manganese, sodium, and potassium. In this context, studies demonstrate a direct correlation between increased CVDs incidence and inadequate intake of some micronutrients. In this regard, review studies showed that anemia is an independent predictor of death among individuals experiencing coronary artery disease, heart failure (HF), and pulmonary hypertension^37,38^. Additionally, a systematic review study demonstrated that iron deficiency is linked to a higher risk of CVDs^39^. Because iron is thought to be a key regulator in thrombopoiesis, iron deficiency has been linked to an increased risk of thrombosis and, consequently, a higher probability of CVDs^39,40^.

Another review study found that zinc has an important impact on the occurrence of CVDs^41^. Indeed, intracellular zinc is essential for the redox signaling pathway, in which certain triggers can, like ischemia, can result in the zinc release from proteins and subsequent myocardial damage, and since replenishing zinc can enhance cardiac function and prevent additional damage, it may possess a protective effect on CVDs development^41^. Additionally, zinc aids in the survival of cardiac stem cells, which are crucial for the healing of heart damage, and stops the development of inflammatory processes that arise after myocardial damage^41^. Similar findings involving zinc and CVDs were found in another review^42^. According to this research, because oxidative stress is one of the major risk factors for the onset and progression of CVDs and because zinc is crucial for regulating cell oxidant/antioxidant balance as well as in signaling molecules, a deficiency in this mineral can raise the risk of developing CVD^42^.

This also holds for other minerals. For instance, a review research reveals a causal link between magnesium insufficiency and CVDs prevalence^43^. Indeed, magnesium deprivation promotes progressive vasoconstriction of the coronary vessels, significantly decreasing oxygen delivery and nutrient intake to cardiac myocytes. This problem may be a major factor in the development or progression of CVDs^43^. Additionally, magnesium deprivation can lead to an increase in intracellular calcium concentration, the production of oxygen radicals, and an increase in inflammatory processes in cardiac cells, all of which enhance the risk of CVDs^43^.

The prevalence of CVDs and the calcium deficiency have been the subject of some investigations^44^. Adequate calcium consumption can reduce the risk of CVDs through mechanisms such as promoting sodium excretion and preventing salt retention (50). A potential reason for how serum calcium may safeguard cardiovascular health is the improvement in blood coagulation and arterial stiffness, both of which are connected to this mineral levels^45^. Another research revealed that in order to gain advantages from calcium's protective effect against CVDs, it is advised to consume foods containing calcium instead of taking calcium supplements^46^. This is because taking calcium supplements has been shown to increase the mortality rate from CVDs in some individuals^46^. Consuming enough calcium-rich meals can help the body prevent calcium insufficiency and benefit from this mineral's advantageous function of preventing the development of CVDs.

Potassium is one of the other elements whose sufficient intake can lead to a preventive effect in CVDs. Compared to other electrolytes, the proportion of sodium to potassium in the diet or urine may be a stronger predictor of cardiovascular outcome^47^. Thus, it is advised that even at normal serum potassium concentrations, it is preferable to acquire enough potassium from the diet enabling this mineral to fulfill its activities efficiently due to the potential effects of potassium in the prevention of CVDs^48^. In addition to potassium, a study that examined the connection between manganese and CVDs revealed that this element's deficiencies might dramatically enhance the risk of CVDs^49^. According to this paper, decreasing endothelial dysfunction and oxidative stress with appropriate manganese intake may be the most significant factor in lowering CVD patients' mortality rates^49^.

Other nutrients like copper and selenium, if not consumed sufficiently, can worsen the risk of CVDs. Selenium is a crucial trace element that is involved in numerous metabolic processes. Selenium can play a preventive effect in the development of CVDs since one of its functions is protecting against oxidative stress^50^. Additionally, selenium in Selenoproteins is regarded as a potent antioxidant that plays a variety of activities, including mediating thyroid hormones and controlling calcium flux, which may contribute to heart health^50^. Moreover, copper is crucial for preventing CVDs. A copper-dependent enzyme, superoxide dismutase, is crucial in the body's defense against free radicals. A drop in copper levels in the heart and other organs and increased plasma cholesterol are also brought on by an inadequate amount of copper intake^51^. Impaired regulation of blood pressure and electrocardiograms, plus impaired glucose tolerance, are also linked to copper deficiency^51^. Therefore, it appears that consuming a diet high in copper may play a preventative role in the development of CVDs.

### Limitations and strengths

The large sample size included in this study is one of its benefits. Additionally, we evaluated the overall dietary pattern of the study population, as opposed to investigations that contrast the association between predefined dietary scores (including the Mediterranean diet or Healthy Eating Index) and non-communicable diseases. Our research has shown that it is possible to have a better understanding of the relationship between dietary habits and non-communicable diseases like CVDs by evaluating both nutritional quality and overall quantity. Adopting a validated and acceptable FFQ for the target population is another benefit of this research.

Notwithstanding these strengths, there are limitations in our research. Since the FFQ data are gathered retrospectively, the recall error in the target population should be considered. Despite the fact that the sizes of the United States Department of Agriculture (USDA) were used to calculate the standard size of each food, there is still some probability of under and over-reporting in our investigation. It should be mentioned that because this research was carried out among residents of a specific geographic area, it is possible that the results cannot be applied to other regions of the world. It was not able to demonstrate a cause-effect relationship in this research due to its nature; hence it is recommended to conduct clinical trials in prospective studies in the target group to better comprehend this problem. It is recommended that future research take into account measuring body composition in order to better understand this issue. In order to completely understand the connection between dietary pattern and non-communicable diseases, it is recommended that a longitudinal study in this field be carried out in the future.

## Conclusion

This study, to our knowledge, was the first to assess the relationship between the prevalence of CVDs in Iran and the latent profile of dietary intake. According to the findings of our study, there is a 1.3-fold increase in the risk of CVDs for people consuming fewer servings of all food groups, both healthy and unhealthy. Additionally, we noticed that individuals in the Low-intake group were in a lower SES class and engaged in much less physical activity, which may have contributed to the greater CVDs prevalence in this group. In addition, we witnessed that the Low-intake group consumed substantially less fiber and minerals than the High-intake group, which may predispose individuals in this group to malnutrition, contributing to the greater CVDs prevalence. A more accurate forecast concerning overall dietary patterns and the prevalence of NCDs can be made by evaluating general dietary patterns as opposed to using a specific diet or food. This problem not only aids the individual in avoiding or better controlling NCDs, but it also aids health policymakers in lowering the prevalence of chronic illnesses like CVDs in society by altering the predominant dietary pattern. To better understand the relationship between the latent profile of dietary intake and the incidence of CVDs, it is recommended to conduct longitudinal studies in the future.

## Materials and methods

The population of the Fasa Adults Cohort Study (FACS) was employed to gather the data for this cross-sectional study. In order to assess the risk factors of NCDs in the city of Sheshdeh and its 24 covered villages (Fasa, Iran), the FACS longitudinal study was carried out. It is also a part of the Iranian prospective epidemiological studies (PERSIAN)^52^. Over 10,000 individuals between the ages of 35 and 70 have signed up for this research.

According to the most recent census, about 250,000 people live in Fasa City, situated in the southwest of Iran and the eastern part of the province of Fars. Sheshdeh rural region (28°56′56.0′′ N, 53°59′26.9′′ E), which has a population of roughly 41,000, was chosen for this study. It was decided to select individuals between the ages of 35 and 70 for this investigation since they are old enough and exposed to health threats and risk factors for NCDs, but they are young enough not to be exposed to final complications of CVDs and other NCDs.

In this investigation, registration was initiated using the registration of the national code (IDID), and subsequently, the individual was assigned a unique code (PCID), which was to be employed in all questionnaires and forms. It should be noted that all participants must fill out an informed consent form at the start of the study. The food frequency questionnaire (FFQ) and general information should be filled out electronically for this study to increase precision and validity. All participants' heights and weights were then assessed by a Bioelectrical impedance analysis device (Tanita BC-418, Tanita Corp, Japan), and their body mass index (BMI) was determined by dividing their weight (Kg) by the square of the height (m^2^). It is noteworthy mentioning the main study protocol agreed with the Helsinki Declaration. The protocol of the study also was approved by the Institutional Review Board (IRB) of Fasa University of Medical Sciences (FUMS) (IR.FUMS.REC.1402.092).

### Food frequency questionnaire (FFQ)

A modified 125-item FFQ was employed to assess the eating behaviors and types of food consumed by the participants over the course of the previous year. This questionnaire is an illustration of Willett's formats^53^, with the essential adjustments made in accordance with Iranian foods. Participants were questioned about their daily, weekly, monthly, and annual consumption of each of the listed foods (Iranian foods) over the previous year. The United States Department of Agriculture (USDA) measurements were used to establish the typical size of each food. Then, the values gathered for each food were translated into grams per day. Micronutrients like calcium, magnesium, zinc, copper, manganese, selenium, sodium, potassium, and iron were also measured. The quantity of total fiber was additionally measured in the current investigation. It should be emphasized that the above-mentioned minerals, as well as the quantity of energy and total fiber, were measured using Nutritionist IV software (version 7.0)^54^. Using this software, the amount of minerals was estimated by multiplying the consumption frequency of each food item by the nutritional content and then summing across all items. More details are accessible at ncdrc.fums.ac.ir/collaboration.

Ultimately, the data from the FFQ questionnaire were separated into 30 groups based on nutritional similarities (Supplementary Table No. 2), and the consumption of each group was given in grams per day in order to better understand the latent dietary profile. The final stage involved breaking down these 30 groups into five categories: whole grains and refined grains; vegetables, fruits, and legumes; red meat and processed foods; oil and fat groups; and ungrouped items. Because of minimal consumption, it should be highlighted that alcohol use was not reported. Participants whose energy intake was outside the usual range (500–3500 kcal/d for women and 800–4000 kcal/d for men) (n = 1819) were excluded from the current investigation to decrease the effect of measurement error^7^. In the end, 8319 individuals were included in the study, and their eating pattern were analyzed.

### Measurement of other indicators

To estimate the prevalence of CVDs in the study population, accurate records of patients' histories of CVDs were made, including myocardial infarction (MI), coronary heart disease (CHD), stroke, or any hospitalization or medication as a result of heart disease^54^. The group with one of the above conditions was identified as the group suffering from CVDs. The metabolic equivalent score (Met) was reported using the International Physical Activity Questionnaire (IPAQ) which was employed to quantify physical activity^54^.

A demographic questionnaire was used in this study to examine variables like age, sex, smoking, and use of other drugs. The Socioeconomic Status (SES) index was estimated using household assets. Since it is highly challenging to get full information on people's income levels, asset data are typically employed in developing countries. Assets including home ownership or rental, house area, number of rooms, presence or absence of a bathroom, refrigerator, freezer, microwave, washing machine, dishwasher, vacuum cleaner, computer, laptop, television (black-and-white, color, LED and LCD), mobile phone, motorcycle, car or truck, and home Internet access were assessed in this study^55^. Ultimately, to analyze this index, it was grouped into three groups, and each category incorporates one-third of the specified properties. In this regard, the Low index contains the poorest individuals, while the High includes the richest ones.

### Statistical methods

In this investigation, individuals were clustered according to their dietary intake using latent profile analysis (LPA). The goal of LPA, a method for classifying latent variables, is to find latent subgroups in a population based on a certain set of variables. Therefore, LPA assumes that participants can be categorized into groups with varying probabilities based on personal or environmental factors^56^. Indeed, LPA establishes a link between a group of continuously observed indicators and a group of latent profiles. LPA and Latent Class Analysis (LCA) differ in the use of variables type. Quantitative variables are used in LPA, while categorical variables are used in LCA^56^.

LPA, like Confirmatory Factor (CFA), uses the covariance matrix to identify latent structures; however, LPA uses the covariance matrix of participants to identify latent groups of individuals, whereas CFA uses the covariance matrix of items to identify latent structures. The fundamental distinction is that CFA analyzes covariances to demonstrate connections between variables, while LPA analyzes covariances to demonstrate connections between individuals^57^.

The LPA model is formulated as follows:

$$\sigma_{i}^{2} = \sum\limits_{k = 1}^{K} {\pi_{k} } \left( {\mu_{ik} - \mu_{i} } \right)^{2} + \sum\limits_{k = 1}^{K} {\pi_{k} \sigma_{ik}^{2} } ,$$

where, µ_ik_ and σ_ik_ stand for the variable's (i) mean and variance (k), respectively, and π_k_ indicates the profile's density or the percentage of participants belonging to k profile.

The main objective of LPA is to identify latent profiles or groups (k) of participants (i) with a significant and understandable pattern of response to interested criteria (j). Common and marginal probability is employed in both intra-class and inter-class models in this study. The intra-class model is defined as follows:

$$\begin{aligned} y_{ij} & = \mu_{j}^{\left( k \right)} + \varepsilon_{ij} \\ \varepsilon_{ij} & \sim N\left( {0,\sigma_{j}^{2\left( k \right)} } \right) \\ \end{aligned}$$

where, ${\mu }_{j}^{(k)}$ represents the mean and ${\sigma }_{j}^{2(k)}$ represents the variance, which varies across all replies for classes or profiles where j = 1 to J and K = 1 to K. According to the general assumptions of LPA, the response variables in each class have a normal distribution and are independent of one another within each class. The non-normality of the response variable can, nevertheless, to some extent be disregarded because this model is a part of mixed models^57^. In this research, the variables utilized in the LAP analysis were normalized using the "logarithm" transformation.

The inter-class model expresses the likelihood of belonging to or being assigned to a certain class k:

$$p\left( {c_{i} = k} \right) = {{\exp \left( {\omega^{\left( k \right)} } \right)} \mathord{\left/ {\vphantom {{\exp \left( {\omega^{\left( k \right)} } \right)} {\sum\limits_{k = 1}^{K} {\exp \left( {\omega^{\left( k \right)} } \right)} }}} \right. \kern-0pt} {\sum\limits_{k = 1}^{K} {\exp \left( {\omega^{\left( k \right)} } \right)} }}$$

where, c_i_ represents the individual's latent classification variable and ${\omega }^{(k)}$ represents a polynomial intercept, fixed at 0 for the final class.

As a result, using the total law of likelihood, the intra-class and inter-class models can be integrated into one model, which raises the following equation:

$$f\left( {{\varvec{y}}_{i} } \right) = \sum\limits_{k = 1}^{K} {p\left( {c_{i} = k} \right)} f\left( {{\varvec{y}}_{i} \left| {c_{i} = k} \right.} \right)$$

where, the likelihood of placement in a class or profile was used to weight the final probability density function for an individual (i) after adding the common inter-class density probabilities for j response variables.

An individual's posterior probability, which is defined as follows, is the result of the LPA analysis.

$$t_{ik} = p\left( {c_{i} = k\left| {{\varvec{y}}_{i} } \right.} \right) = \frac{{p\left( {c_{i} = k} \right)f\left( {{\varvec{y}}_{i} \left| {c_{i} = k} \right.} \right)}}{{f{\varvec{y}}_{i} }}$$

According to their scores in the responses variables in the vector y_i_, it shows the likelihood of placing a person (i) in a class (c_i_) or a certain profile (k).

For each person in each profile, a posterior probability (t) is determined, with values closer to 1 demonstrating a higher likelihood of membership in that particular profile. There is more certainty regarding a person's membership allocation as the difference between the posterior probability for that person increases^57^. Different models were fitted to choose the best one for this analysis, and the best model was chosen after comparing its properties, including Akaike information criterion (AIC), Bayesian information criterion (BIC), entropy, and interpretability.

To report independent variables based on latent classes, frequency and percentage for qualitative variables and mean and standard deviation for quantitative variables were employed after calculating the number of classes. The variables in the latent class subgroups were also compared using a one-way analysis of variance and the chi-square test. Due to violating the proportional odds assumption, multinomial logistic regression was used to determine the association between covariates and latent classes. The type one error (α) was set to 0.05 in each analysis.

### Ethical approval and consent to participate

The study protocol was following the Helsinki Declaration and was confirmed by the Ethics Committee of Fasa University of Medical Sciences (Approval Code: IR.FUMS.REC.1402.092). The participants were informed about the research objectives and a consent form through online was obtained from the subjects before starting the survey.

### Supplementary Information

Supplementary Tables.

## Data Availability

The datasets used and/or analyzed during the current study are available from the corresponding author on reasonable request to the corresponding author.
