# Supplementary material for: Association between latent profile of dietary intake and cardiovascular diseases (CVDs): Results from Fasa Adults Cohort Study (FACS)
Source: Sci Rep. 2023 Oct 18;13:17749. doi: 10.1038/s41598-023-44766-4 (PMC10584898; doi:10.1038/s41598-023-44766-4)
Supplement: Supplementary file 1 — Supplementary Tables. [file 41598_2023_44766_MOESM1_ESM.docx]

Supplementary Table 1. Baseline characteristics of the study population based on gender

| **Categorical measurement** | Subgroup | Male | Female | p-value |
| --- | --- | --- | --- | --- |
|  |  | N (%) * | N (%) * |  |
| Socio Economic Status (SES) | Low | 1020(26.25) | 1753(39.57) | <0.001** |
|  | Medium | 1318(33.92) | 1453(32.80) |  |
|  | High | 1548(39.84) | 1224(27.63) |  |
| Smoking | No | 3321(85.44) | 4307(97.18) | <0.001** |
|  | Yes | 566(14.56) | 125(2.82) |  |
| Use Drugs | No | 2115(54.44) | 4394(99.16) | <0.001** |
|  | Yes | 1770(45.56) | 37(0.84) |  |
| Having Cardiovascular Disease | No | 3514(90.47) | 3814(86.08) | <0.001** |
|  | Yes | 370(9.53) | 617(13.92) |  |
| **Continues measurement** | **-** | **Mean ±SD** | **Mean ±SD** | **p-value** |
| Age | - | 48.65± 9.65 | 48.84± 9.54 | 0.363 ^***^ |
| Energy intake | - | 2690.002± 734.79 | 2417.415± 628.94 | <0.001^***^ |
| Physical activity (MET) | - | 45.20± 14.30 | 38.26± 6.64 | <0.001^***^ |
| BMI | - | 24.19± 4.43 | 26.83± 4.87 | <0.001^***^ |

*Within column relative frequencies

**p-value for chi-square test

***p-value for independent sample t-test

Abbreviations: BMI—body mass index

Supplementary Table 2. Food groupings used in the dietary profile analysis

| Food groups Food items | |
| --- | --- |
| Whole grains versus Refined grain | |
| Whole grains | Cooked whole barley/wheat, Wholegrain bread, oatmeal, and other wholegrain products |
| Refined grains | All types of White bread, rice white, cooked pasta/lasagna, bagels/sweets, muffins/biscuits/cakes |
| Vegetables, fruits and legumes | |
| legumes | Beans or lentils, tofu soya protein, peas or lima beans, cotyledons, Broad bean |
| Leafy vegetables | stewed vegetables, Spinach cooked, spinach raw, head lettuce, leaf lettuce |
| Crucifeerois vegetables | Cabbage (including white, red, cauliflower and broccoli, Brussels sprouts, kale), Raw or cooked celery/artichoke, mustard/chard greens |
| Tomatoes | Tomatoes, tomato sauce |
| Other vegetables | cucumber, Onions raw, onions cooked, Bell pepper / green pepper, corn, mixed vegetables, cooked mushrooms, Green Peas, Green beans, Local Vegetables |
| Dark yellow vegetables | Carrots raw, carrots cooked, squash, Stewed eggplant, yams/sweet potatoes, Beetroot/turnip |
| Potatoes | Potatoes (boiled and fried) |
| Summer spring fruits | Cantaloupe, Melon, watermelon, Apricot, Cherry, Peach, Greengage, fresh berries (white, red, blackberry), Strawberries, plums (yellow and red), fresh figs, mango, Green Almond |
| Winter autumn fruits | grape, pear, Citrus (oranges, tangerines, sour lemons, limes, grapefruits), Pomegranate, Persimmon |
| All season fruits | Apple, Kiwi, banana, date, Dry fruits (figs, peaches, plums, apricots), Raisins, currants, berries |
| Red meat and processed foods | |
| Red meat | Red meat (boiled, stewed, minced, grilled), beef/lamb |
| Processed meats | Sausages, Bacon/beef hot dogs, chicken/turkey hot dogs, regular hamburgers, and other processed meat |
| Pizza | Pizza, Pizza cheese |
| French fried | French fries, Potato chips |
| Snacks | crackers, crackers whole grain, popcorn light, cheese puffs |
| Sweets | Candies, local sweets, Creamy dessert / Creamy cake, Chocolates/breakfast chocolates, candy bars |
| Mayonnaise and creamy dressing | Mayonnaise regular, salad dressing |
| Oil and fat groups | |
| Butter/margarine/oil | Butter, spreadable butter, margarine, animal oil, Liquid/vegetable oil |
| Olive oil | Olive oil, olive |
| Nuts | walnut, peanuts, other nuts (almonds/cashews/pistachios/hazelnuts), Seeds (pumpkin, sunflower, watermelon) |
| Ungrouped foods | |
| Poultry | Chicken/turkey, other Poultry |
| Organ meal | Liver beef/ sheep, liver chicken/turkey, other chicken parts (heart, gizzard), sheep's head and trotters |
| Eggs | Eggs regular, egg whites |
| Fish and seafood | Tuna canned, all types of fish |
| Low and High-fat dairy | Milk, yogurt, cheese, Dough, Curd, colostrum, cream, regular ice cream |
| Fruit juice | Lemon juice, orange juice, All kinds of other juice |
| Condiments | jams/preserves/honey, salt added, sugar Loaf, number of teaspoons of sugar, all Types of pickles, Pickled cucumber, ketchup/red chilli sauce |
| Tea | Tea with caffeine, decaffeinated tea |
| Energy drink | Soft drinks/soda, malt beer, Industrial juice, sugar beverages other |
| Coffee | Coffee with caffeine, decaffeinated coffee |
